# Creating and testing a GCP game in an asynchronous course environment: The game and future plans

**DOI:** 10.1017/cts.2019.423

**Published:** 2019-10-29

**Authors:** Carolynn T. Jones, Penelope Jester, Jennifer A. Croker, Jessica Fritter, Cathy Roche, Brian Wallace, Andrew O. Westfall, David T. Redden, James Willig

**Affiliations:** 1 Masters of Applied Clinical and Preclinical Research, College of Nursing, The Ohio State University, Columbus, OH, USA; 2 Center for Clinical and Translational Science, University of Alabama at Birmingham, Birmingham, AL, USA; 3 School of Medicine, University of Alabama at Birmingham, Birmingham, AL, USA; 4 Nationwide Children’s Hospital, Columbus, OH, USA; 5 School of Nursing, University of Alabama at Birmingham, Birmingham, AL, USA; 6 Department of Biostatistics, School of Public Health, University of Alabama at Birmingham, Birmingham, AL, USA

**Keywords:** Gamification, GCP training, clinical research, clinical research professionals, good clinical practices

## Abstract

**Introduction:**

The National Institute of Health has mandated good clinical practice (GCP) training for all clinical research investigators and professionals. We developed a GCP game using the Kaizen-Education platform. The GCP Kaizen game was designed to help clinical research professionals immerse themselves into applying International Conference on Harmonization GCP (R2) guidelines in the clinical research setting through case-based questions.

**Methods:**

Students were invited to participate in the GCP Kaizen game as part of their 100% online academic Masters during the Spring 2019 semester. The structure of the game consisted of 75 original multiple choice and 25 repeated questions stemming from fictitious vignettes that were distributed across 10 weeks. Each question presented a teachable rationale after the answers were submitted. At the end of the game, a satisfaction survey was issued to collect player satisfaction data on the game platform, content, experience as well as perceptions of GCP learning and future GCP concept application.

**Results:**

There were 71 total players who participated and answered at least one question. Of those, 53 (75%) answered all 100 questions. The game had a high Cronbach’s alpha, and item analyses provided information on question quality, thus assisting us in future quality edits before re-testing and wider dissemination.

**Conclusions:**

The GCP Kaizen game provides an alternative method for mandated GCP training using principles of gamification. It proved to be a reliable and an effective educational method with high player satisfaction.

## Introduction

Good clinical practice guidelines (GCPs) are a broad set of recommendations and requirements that have been developed to assure the ethical conduct of research, increase the protection of human participants, and to ensure collection of credible, rigorous, and reproducible data. The International Council on Harmonization of Technical Requirements for Pharmaceuticals for Human Use (ICH) has existed for 25 years, focusing global pharmaceutical regulatory harmonization in a centralized venue for best practices in medical product development and research. The harmonized guidelines are divided into four categories: Quality, Safety, Efficacy, and Multidisciplinary. Found within the Efficacy Guidelines, Section 6 (R2), the GCPs are internationally accepted guidelines describing responsibilities and best practices in accordance to the Declaration of Helsinki (*ICH E6 (R2)*). In September 2016, National Institute of Health (NIH) determined that all NIH-funded investigators and staff involved in the conduct, oversight, or management of clinical trials must be trained in GCP, and this became an official NIH mandate in January 2017 (https://grants.nih.gov/grants/guide/notice-files/not-od-16-148.html). Most institutions, Institutional Review Boards (IRBs), and clinical trial sponsors have adopted this requirement as policy, regardless of funding source. In addition to federal laws and local institutional policies, ICH GCPs are key guidelines that sponsors, investigators, and clinical research staff should know and follow to ensure study safety, efficiency, and reliability. Notably, the Clinical Trials Transformation Initiative developed recommendations for GCP training practices, including an emphasis on exploring methods for case-study approaches to help learners incorporate application of GCP requirements in study activities [1]. Training opportunities for certifying clinical research and GCPs training are offered by the CITI Program (citiprogram.org), available through individual and institutional subscriptions.

Gamification refers to the introduction of game mechanics into other milieus to enhance user engagement. Educators use gamification to enhance student engagement with and learning of course content. To gamify teaching and learning materials, gamification elements are introduced as the mechanism to deliver content and motivate learners. Learners are motivated to engage with the game, and with peers, through a series of intrinsic (self-efficacy, personal challenge of problem solving, etc.) and extrinsic motivators (acquisition of points, levels and rewards, and team competition) [2–5].

The purpose of this paper is to describe and evaluate GCP Kaizen, a GCP learning method utilizing internet-based gamification to supplement required GCP training. Developed by the Center for Clinical and Translational Science program at the University of Alabama at Birmingham (UAB), the Kaizen-Education software platform provides online game manager tools to develop multiple-choice questions and schedule those questions within games as well as mobile- and computer-based interfaces to play the games. The Kaizen-Education software platform is named after the Japanese concept of continuous incremental process improvement, a concept analogous to the higher education goal of creating lifelong learners. Several games have been developed using this system, including games aimed at reinforcing graduate medical education in internal medicine and otolaryngology [6,7]. The Kaizen-Education software platform was similarly adopted by the UAB School of Nursing for undergraduate nursing courses [8–10]. Additional uses of the Kaizen-Education software platform have examined player competencies in public health and supported formal training of rigor, reproducibility, and transparency. To date, games in Kaizen have been developed and used by 16 institutions and across 13 states. The GCP Kaizen game was produced collaboratively by UAB and The Ohio State University (OSU) Centers for Clinical and Translational Science through the following grants from the National Center for Advancing Translational Sciences (NCATS), National Institutes of Health: UL1TR001417-03S1; UL1TR002733. The GCP Kaizen game was designed to help clinical research professionals immerse themselves into applying ICH GCP (R2) guidelines in the clinical research setting.

## Materials and Methods

### Setting

We created the GCP Kaizen game to provide a fun and applicable gamification approach to learning GCPs. This paper details the construction of the game and evaluation of the game quality using item analysis. We also report measures of player satisfaction and propose opportunities to improve future games based on player recommendations.

A series of brief, fictitious vignettes followed by multiple-choice game questions formed the structure of the game. A total of 75 original and 25 repeat questions were included in the game to measure knowledge retention. The game was tested in two graduate clinical research courses offered at OSU during the Spring 2019 semester: one in its Masters of Clinical Research program and another in the Masters of Science in Clinical and Translational Pharmacology. This study project was determined exempt by OSU IRB. Case studies and associated questions were drafted by the course instructors (CJ and PJ) and reviewed by an external consultant (CR). Once questions and associated illustrations and rationales were completed, question content and reward parameters were uploaded to the Kaizen platform and scheduled for release. Table 1 illustrates the frequency of GCP topics covered in the game.

**Table 1.** Frequency of GCP topics in questions

Frequency of topics		
Regulatory guidance document/topic	Questions 1–75	Repeat questions 76–100
Belmont Report and Declaration of Helsinki	6	2
ICH E6 R2 (Introduction, Section 1 and 2)/Introduction, Glossary, and GCP Principles	10	6
ICH E6 R2 (Section 3)/Institutional Review Board (IRB)	7	2
ICH E6 R2 (Section 4)/Investigator Responsibilities	18	2
ICH E6 R2 (Section 5) / Sponsor Responsibilities	16	6
ICH E6 R2 (Section 6)/Protocol and Protocol Amendments	7	1
ICH E6 R2 (Section 7)/Investigator Brochure	5	3
ICH E6 R2 (Section 8)/Essential Documents	6	3

GCP, Good clinical practice guidelines; ICH E6 R2, International Conference on Harmonization, Efficacy Guidelines #6, Revision 2; IRB, Institutional Review Board.

Players were provided a document titled “Welcome Overview and Informed Consent” for instructions and consent for participation in the game and a final game evaluation survey via emails from the study team and through OSU’s online Learning Management Platform, Canvas^®^ (Instructure) Course Announcements. At registration for game participation, players used their campus email address and were provided a standardized game password. They were instructed to create an alias, for privacy purposes. The invitation to participate in the game included prestudy links to associated GCP documents, and participants were advised to review the documents prior to answering the Kaizen questions. In addition to providing links to the ICH E6 (R2) GCPs, we also provided links to the Belmont Report [11], Declaration of Helsinki [12], Title 21 Code of Federal Regulations, Part 312 [13], and a handout about effective and efficient monitoring for quality assurance [14].

### Games Structure

Players were informed that game participation was voluntary and that data gathered from the game would not affect their academic course scores or class standing. The game was comprised of 100 total questions that were released on a schedule of 10 questions per week, for 10 weeks. We estimated that it would take players up to 20 minutes to answer 10 questions. Weekly email announcements were issued about the release of new questions and to remind players to participate. Additionally, the course calendar also included reminders to complete game questions. Another motivating strategy included reports about game progress, individual status, and team ranking, which were sent to players via the online course announcements/email mechanism. Players also had access to leaderboards when they signed onto the Kaizen-Education software platform to answer questions which displayed their individual and team rankings in real time.

Since the ICH GCPs are “internationally accepted,” we designed an Olympic Games-inspired theme to provide in-game rewards. Teams were named by Country. Northern Hemisphere countries represented team names for one course and Southern Hemisphere countries represented teams for the second course. Players were assigned to teams by their instructors. Players from the two courses participated in the game.

Reward badges and points were automatically issued to players based on predetermined participation and answer thresholds, and these data were used to populate the leaderboard. Level badges were issued at 20-point increments based on total points accrued. Hot Streak badges awarded for consecutive right answers were issued for at 15, 20, and 25 correct questions in a row.

### Item Analysis

All player interactions with the Kaizen-Education software platform, including number of questions answered, responses, and timing of answers are captured by the software database. The data captured by the Kaizen-Education software platform were used to complete the analyses below.

To access question difficulty, the proportion of students answering correctly was calculated per question for the 75 original questions [15]. To measure internal consistency and estimate how well the questions reliably measure knowledge of GCP, Cronbach’s alpha was estimated. Cronbach’s alpha is a measure of the internal consistency of test items, with a score ranging from 0 to 1. A high Cronbach’s alpha ranging from 0.70 to 0.95 suggests a high degree of internal consistency [16].

Point biserial correlations range from −1 to 1. A correct interpretation of point biserial correlation is that it compares the average total score from the game for all players who answered that specific question correctly to the average total score from the game for all players who answered that specific question incorrectly. To avoid overestimation of the point biserial correlation, it is common to adjust the total scores by removing the points earned from the question under investigation. A positive point biserial correlation indicates the highest scoring players answered the question correctly while the lowest scoring players answered the question incorrectly [15]. Therefore, the question discriminates high performers from low performers. A negative point biserial correlation indicates poor quality by indicating the highest performers missed the question while the lowest performers answered correctly. Ultimately, point biserial correlations can be used to reveal questions that may benefit from review and editing. A strong point biserial correlation is considered to be ≥0.2 [15].

### Knowledge Retention

McNemar’s test was used to compare the paired proportions for the 25 repeated questions to determine if re-exposure to the questions might indicate students who missed the question previously remembered/learned the material and answered correctly, while those who answered correctly the first time maintained the knowledge.

### Player Satisfaction Survey

We issued a postcourse evaluation using Qualtrics^xm^ survey software. The survey asked players about their satisfaction with their experience, including the Kaizen platform, course content and materials, time commitment, as well as perceptions of GCP learning and application. The survey was distributed to all players via course announcement and email a week after the final set of questions was issued. Reminders were sent weekly for 3 weeks.

All analyses were conducted using SAS 9.4 (Cary, NC, USA). Player participation and percentage of questions answered were calculated.

## Results

A total of 71 players participated and answered at least one question. Of those, 53 (75%) answered all 100 questions (75 original GCP, 25 repeats for knowledge retention). A total of 55 (77%) answered all of the 75 original questions. Most players (*n* = 63, 89%) had previously acquired GCP Certificates from CITI. Additional descriptive data are provided in Table 2.

**Table 2.** Player activity/performance data

**Players or teams**	**Course 1**	**Course 2**	**Combined**
Originally enrolled	31	49	80
Completed the academic course	31	47	78
Registered for GCP Kaizen game	31	45	76
Played GCP Kaizen game	29	42	71
Answered all 100 questions	28	25	53
Answered 50–100 questions	3	6	9
Answered up to 49 questions	0	9	9
Number of teams	6	9	15
Number of who completed with a score of at least 151 points (75% correct)	21	23	44
Average score of players	154.10	128.05	138.69

GCP, Good clinical practice guidelines.

### Item Analyses

**Fig. 1.**
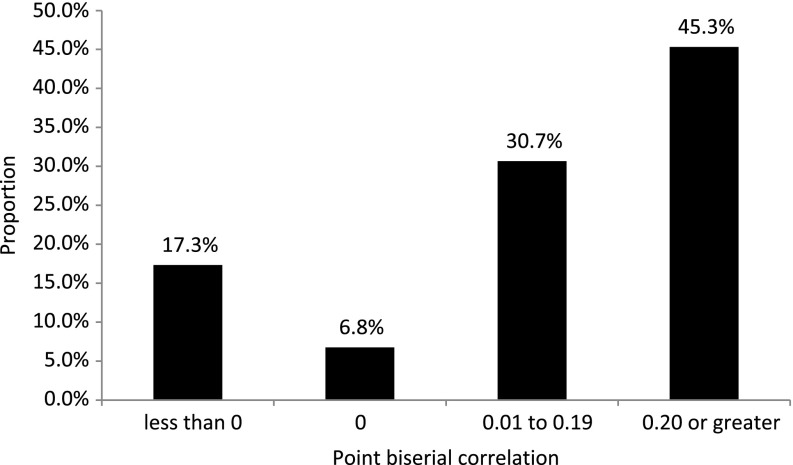
Proportion of questions with point biserial correlations within specified ranges.

**Fig. 2.**
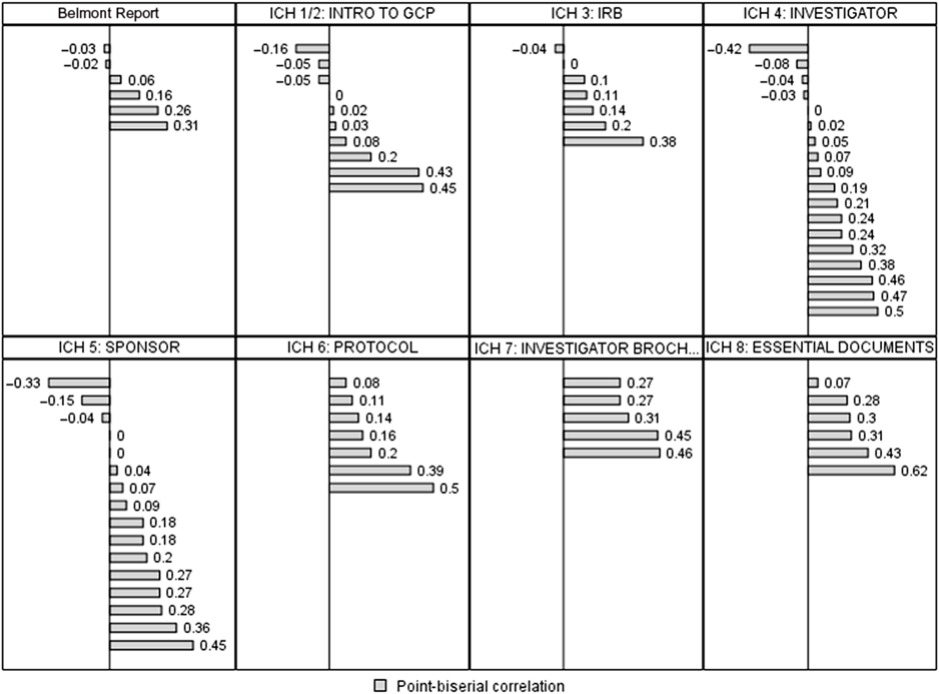
Point biserial correlations by ICH domain. *Note:* GCP, Good clinical practice guidelines; ICH, International Conference on Harmonization; IRB, Institutional Review Board; Intro, introduction.

Cronbach’s alpha for the 75 original questions indicated a reliability of 0.74. Across the 75 original questions (*n* = 55), the percentage of correct answers ranged from 3.6 to 100 (median 83.5). Point biserial correlation (calculated for the 55 individuals who completed the original 75 questions) ranged from a high of 0.61 (6th question asked in module ICH 8: Essential Documents) to a low of −0.42 (the first question asked in module ICH 4: Investigator Responsibilities). The median point biserial correlation for the 75 questions is 0.16 with 83% of all questions either having a point biserial correlation of 0 or greater. The percentage of questions with a point biserial correlation within specified ranges is summarized in Fig. 1. Fig. 2 shows the distribution of point biserial correlation values for all questions pertaining to specific course topics.

### Knowledge Retention

Table 1 shows the number of questions per topic as well as the number of repeated questions to test for knowledge retention reintroduced for each. Comparing responses for the 25 original and their repeated questions revealed an improvement in the proportion answering correctly for some questions, though sample size and ceiling effects limit the statistical power to declare statistically significant increases (Table 3).

**Table 3.** Comparison of original and repeated questions (*n* = 53)

**ICH domain**	**Original % correct**	**Repeat % correct**	***P*** **-value**
ICH Intro	83.0	94.3	0.0339
ICH Intro	77.4	79.2	0.5637
ICH Intro	86.8	90.6	0.5291
Belmont Report	73.6	66.0	0.3458
Belmont Report	92.5	92.5	1.00
ICH Section 2 (Principles of GCP)	86.8	88.7	0.7630
ICH Section 2 (Principles of GCP)	66.0	84.9	0.0047
ICH Section 2 (Principles of GCP)	62.3	62.3	1.00
ICH Section 3 (IRB)	94.3	92.5	0.6457
ICH Section 3 (IRB)	88.7	81.1	0.1573
ICH Section 4 (Investigator)	88.7	75.5	0.1088
ICH Section 4 (Investigator)	52.8	74.4	0.0046
ICH Section 5 (Sponsor)	100.0	98.1	–
ICH Section 5 (Sponsor)	66.0	71.7	0.3657
ICH Section 5 (Sponsor)	90.6	94.3	0.3173
ICH Section 5 (Sponsor)	71.7	69.8	0.7815
ICH Section 5 (Sponsor)	7.5	32.1	0.0008
ICH Section 5 (Sponsor)	92.5	92.5	1.00
ICH Section 6 (Protocol)	67.9	79.2	0.1088
ICH Section 7 (Investigator Brochure)	88.7	83.0	0.3173
ICH Section 7 (Investigator Brochure)	84.9	77.4	0.2059
ICH Section 7 (Investigator Brochure)	86.8	88.7	0.7630
ICH Section 8 (Essential Documents)	98.1	96.2	0.5637
ICH Section 8 (Essential Documents)	66.0	83.0	0.0126
ICH Section 8 (Essential Documents)	90.6	90.6	1.0

The data in Table 3 are from the item analyses of the full set of questions 1–100, with 53 participants completing all 100 items. GCP, Good clinical practice guidelines; ICH, International Conference on Harmonization; IRB, Institutional Review Board.

### Player Satisfaction Survey

**Fig. 3.**
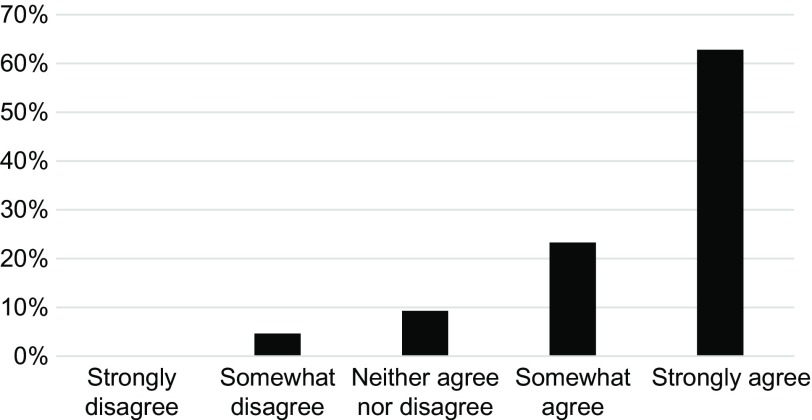
Player perceptions of good clinical practice guidelines knowledge improvement.

Of the 71 players, 44 (62%) responded to the survey. Unanimously (100%), respondents felt that 10 Kaizen questions per week were reasonable and were somewhat satisfied (23%) or very satisfied (68%) with the ease of use of the GCP Kaizen questions. Most (89%) stated that they viewed the leaderboard as a player and team member. In terms of overall satisfaction with the GCP Kaizen game, 34% were somewhat satisfied and 55% were very satisfied. When asked if the GCP Kaizen game improved their knowledge of GCP applications, most strongly agreed (Fig. 3).

We asked for qualitative feedback on what they liked the most about the content of the GCP Kaizen game. Many commented that the game made ICH GCP learning fun.

I love trivia, so this was a really fun way to learning about GCP. I also think it is a good way to apply knowledge using situation-based questions.

Players liked the case-based scenarios that preceded each question.

I liked the real-world scenarios the best in the GCP Kaizen game. It is easy to learn facts in a class, but analyzing a real-world situation and what should be done is an application that makes it more easy to translate our knowledge when we go into the clinical research world.

It really helped me learn critical thinking skills and took the lessons learned out of the textbook atmosphere that other courses operate in.

Real-world examples are the most effective to me.

Provided real-life scenarios that simulate hands-on learning. Also liked the explanations to the answers because I learned from those in a ‘fun’ setting.

The progression of individual questions within the same “story” or scenario constructs.

Players also liked that each question included a rationale, to reinforce the learning.

The individual focused questions that gave clear answers upon submission with specific references to ICH E6 (R2).

I liked that you instantly got answers to questions that were correct.

One player completed the internationally recognized Association of Clinical Research Professional (ACRP) Clinical Research Coordinator Certification exam toward the end of the semester and liked that the game provided her with a sound GCP review and contributed to her passing the exam.

I felt like this was a great review of the ICH guidelines and definitely helped me to pass my CCRC certification. I am almost hoping to go back and review the questions so I can help some of my colleagues’ review for the SoCRA CCRP exam as well, because it is great that the guideline number and explanation for each question result came with the answer.

We also asked what they liked most about the competition features of the GCP Kaizen system. Responses affirmed the motivational aspect of individual and team competition.

I liked seeing the leaderboard so I could see how I was doing relative to my teammates and how my team was doing relative to the entire class.

Being able to compare scores made it so I wanted to answer more questions and learn more.

We also solicited criticisms and asked for other general comments. Players reported that some of the questions were too long, they desired hyperlinked references in the rationales, they wanted more than 10 questions per week, and they wanted the ability to move forward on their own timeframe (e.g., having all questions at once).

I am the type of person who would do more than 10 questions at a time, so having a limit isn’t necessary,

Sometimes the wording of the questions was a bit confusing.

I wish that there were links the GCP for a reference to the question.

One player did not like that some questions were repeated. Another player felt that the questions were too “site focused” suggesting that we modify some questions to include other perspectives, such as sponsors and monitor perspectives.

The questions repeated or were very similar. I think that more diverse questions would have helped me learn GCP better.

Several players suggested improved clarity in some of the questions. Finally, some felt that there was a lag in the question loading, though wondered if it was possibly related to their browser speed.

The system moves pretty slow and there is a decent lag time between pressing “go” and “next” between questions.

There was a bit of a lag in the system, but that could have very well been in my browser.

## Discussion

The requirement for documentation of GCP training has been a well-established standard to ensure improved quality, safety, and compliance in clinical research. With the revision of the ICH guidelines to include E6 R2 (Step 4), including the emphasis on risk-based approaches for study development, monitoring, and quality improvement, continued calls for GCP training have been issued, especially to address the needs in Social and Behavioral clinical research and in workforce development [17–19]. We have described an innovative approach to GCP training using the Kaizen-Education gamification platform. The game was tested in two asynchronous online academic courses during a single semester. Our “game” (knowledge competition) had a strong Cronbach’s alpha, and item analysis indicated 24% of our questions produced point biserial correlations that were negative or zero. The ability to self-assess questions through item analysis provided valuable information that will catalyze editing of those questions in the next iteration of the GCP game. Finally, our player satisfaction survey results showed that the game was well received and that students thought favorably of the GCP content, as well as the gamification approach to learning provided by the Kaizen Education software.

The traditional training on GCPs has primarily focused on ICH E6 (R2), for example, “GCP Guidelines.” It has been argued that training should go beyond ICH GCP and include statistical concepts (e.g., intent-to-treat) for improvement in trial performance [20]. In the design of the GCP Kaizen, we primarily focused on ICH GCP E6 R2, Belmont and Declaration of Helsinki. In the future, it would be important to expand higher level offerings that would include ICH guidelines less commonly consulted by standard GCP courses, for example, E7 – Clinical Trials in Geriatric Populations; E8 – General Considerations for Clinical Trials; E9 – Statistical Principles for Clinical Trials; E11A – Clinical Trials in Pediatric Populations; E18 – Genomic Sampling; and Q9: Quality Risk Management. In addition to the practical application of these commonly used guidelines, the topics in these other guidelines are beginning to be included in ACRP Certification exams.

Feedback from players indicated that the GCP Kaizen game provided a welcomed and important review of concepts and increased their knowledge of GCP regardless of experience and prior certification. We dedicated considerable content on investigator and sponsor responsibilities compared to the other topics. An understanding of sponsor roles is important for study operations, but especially when the investigators have sponsor responsibilities (e.g., investigator-initiated studies, or managing multi-center studies as Investigational New Drug or Investigational Device Exemption studies). Despite having previous GCP training, participation in the GCP Kaizen game offered important and applicable remediation.

In studying the quality, reliability, and satisfaction of the GCP Kaizen game, we identified a number of limitations. GCP Kaizen participation was voluntary in the two courses and some players taking a higher course load or for other reasons opted to not participate fully. This fact could have created a selection bias that affected our item analysis results. Moreover, most, but not all, of the players had prior GCP training which may have impacted our item analysis evaluation. It may be interesting to test knowledge acquisition in clinical research novices exclusively. It may be valuable for a future offering of the GCP Kaizen game to correlate game performance to years of experience and prior GCP training, by including those descriptors in initial game items.

A GCP Kaizen Phase II project is underway to review and edit test items based on point biserial scores. We included a cohort of higher scoring volunteer players in the revision process. We are re-running the revised new test in an Autumn 2019 online course to test question revisions using item analyses. Data on player levels of experience will be collected to compare results of novices verses experienced players. We plan to have the GCP Kaizen game available in a new Kaizen-Education mobile application by 2020 with information about access through the NCATS-funded Diamond Portal (https://clic-ctsa.org/diamond).

## Conclusions

The GCP Kaizen game was developed and tested to meet a need to provide an alternative approach for mandated GCP training using principles of gamification. The Kaizen Education software platform was a reliable and effective mechanism for game delivery with high player satisfaction. GCP Kaizen contributed to new learning, even among participants who had previous GCP training certificates. Finalized versions of the game will offer an excellent supplement to GCP learning and application in academic and training settings. Future games could be produced to offer new case-based scenarios, expand the variety of perspectives and roles, and to expose learners to additional ICH content.
